# Variation in the Strength of Blue Mass and Mercurial Ointment

**Published:** 1844-07

**Authors:** 


### PRACTICAL MEDICINE, &c.

Variation in the Strength of Blue Mass and Mercurial Ointment.—From the report of the committee on Mercurial Preparations, of the College of Pharmacy, New York, it appears that the blue mass and mercurial ointment, as prepared by different manufacturers, is subject to a considerable variation. In the former the per centage of mercury was in some specimens as low as 20, in others as high as 34! in the ointment it varied from 21 to 46. The upper stratum of ointment was, in some instances, 4 per cent. less rich in mercury than the lower stratum in the same jar, as the result of settling. As these facts are important to those who have frequent occasion to use these preparations, we extract from the American Journal of Pharmacy the following letter describing the processes used for testing their strength:

"New York, Feb. 13, 1844.

"*Gentlemen:* In compliance with your request, I beg leave to state, that the following methods were adopted to ascertain the per centages of mercury in blue pill and mercurial ungt.

"The blue pill was washed with warm water, and also alcohol, to remove such organic matters as were soluble in these fluids; it was dried until it was of the consistence of a thick paste, then mixed with an equal weight of sulphuric acid,—the whole being heated until the remaining organic matter was decomposed and carbonised; the persulphate of mercury and carbonised matter so obtained were boiled with a solution of chloride of tin, to reduce the mercury; the precipitate was washed and dried, and the mercury separated by sublimation in glass tubes.

"The mercurial ointment was treated with warm water, by which means the greatest part of the lard separated, floating on the water; it was then washed with a little alcohol to remove any water attached to it, and then triturated with hot spirits of turpentine, which displaced the remaining unctuous matters; then washed with warm alcohol twice, to cleanse it from the turpentine and grease: upon drying it at a temperature of about 90 degs. the mercury will assume the form of globules, or may be made to do so by pressing it together.

"Your most obedient servant,

"Lawrence Reid.

"To the Committee of the College of Pharmacy, New York, on Mercurial Preparations."

